# 2′-Iodo-2,2′′,3,3′′,4,4′′,5,5′′,6,6′′-deca­methyl-1,1′:3′,1′′-terphenyl chloro­form monosolvate

**DOI:** 10.1107/S1600536810052736

**Published:** 2010-12-24

**Authors:** Marian Olaru, Sorin Roşca, Ciprian I. Raţ

**Affiliations:** a Universitatea Babeş-Bolyai, Facultatea de Chimie şi Inginerie Chimicã, 11 Arany Janos, 400028 Cluj-Napoca, Romania

## Abstract

The title compound, C_28_H_33_I·CHCl_3_, forms dimers through C—I⋯π inter­actions. The crystal structure is consolidated by the presence of C—H⋯π inter­actions between the chloro­form solvent and the main mol­ecule.

## Related literature

For the synthesis and spectroscopic characterization of 2′-iodo-2,2′′,3,3′′,4,4′′,5,5′′,6,6′′-deca­methyl-1,1′:3′,1′′-terphenyl, see: Hino *et al.* (2005 ▶); Duttwyler *et al.* (2008 ▶). For related *m*-terphenyl iodides, see: Niemeyer (1998 ▶); Twamley *et al.* (2000 ▶); Zakharov *et al.* (2003 ▶). For general background to compounds with *m*-terphenyl substituents, see: Power (2004 ▶).
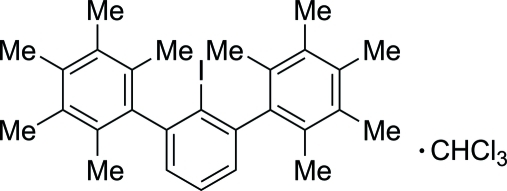


## Experimental

### 

#### Crystal data

C_28_H_33_I·CHCl_3_
*M* *_r_* = 615.81
Monoclinic, 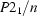

*a* = 12.0294 (10) Å
*b* = 16.0651 (13) Å
*c* = 15.3762 (12) Å
β = 103.385 (1)°
*V* = 2890.8 (4) Å^3^
*Z* = 4
Mo *K*α radiation
μ = 1.40 mm^−1^
*T* = 297 K
0.32 × 0.28 × 0.26 mm

#### Data collection

Bruker SMART CCD area-detector diffractometer
Absorption correction: multi-scan (*SADABS*; Bruker, 2000 ▶) *T*_min_ = 0.663, *T*_max_ = 0.712
22910 measured reflections
5896 independent reflections
4698 reflections with *I* > 2σ(*I*)
*R*_int_ = 0.056

#### Refinement

*R*[*F* ^2^ > 2σ(*F* ^2^)] = 0.066
*wR*(*F* ^2^) = 0.137
*S* = 1.13
5896 reflections
308 parameters
H-atom parameters constrained
Δρ_max_ = 0.83 e Å^−3^
Δρ_min_ = −0.68 e Å^−3^

### 

Data collection: *SMART* (Bruker, 2000 ▶); cell refinement: *SAINT-Plus* (Bruker, 2001 ▶); data reduction: *SAINT-Plus*; program(s) used to solve structure: *SHELXS97* (Sheldrick, 2008 ▶); program(s) used to refine structure: *SHELXL97* (Sheldrick, 2008 ▶); molecular graphics: *DIAMOND* (Brandenburg, 2009 ▶); software used to prepare material for publication: *publCIF* (Westrip, 2010 ▶) and *PLATON* (Spek, 2009 ▶).

## Supplementary Material

Crystal structure: contains datablocks I, global. DOI: 10.1107/S1600536810052736/pk2291sup1.cif

Structure factors: contains datablocks I. DOI: 10.1107/S1600536810052736/pk2291Isup2.hkl

Additional supplementary materials:  crystallographic information; 3D view; checkCIF report

## Figures and Tables

**Table 1:** C—H⋯π inter­actions (Å, °) *Cg*2 and C3 are the centroids of the C7–C12 and C18–C23 benzene rings, respectively.

*D*—H⋯*A*	*D*—H	H⋯*A*	*D*⋯*A*	*D*—H⋯*A*
C26—H26*A*⋯*Cg*2^i^	0.96	3.86 (1)	2.97	155
C28—H28*A*⋯*Cg*2^ii^	0.96	3.53 (1)	2.87	127
C29—H29⋯*Cg*3^iii^	0.98	3.42 (1)	2.44	177

Symmetry codes: (i) 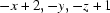; (ii) 
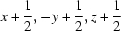
; (iii) 
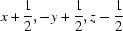
.

**Table 2:** C—I⋯π inter­actions (Å, °) *Cg*3 is the centroid of the C18–C23 benzene ring.

*Y*—*X*⋯*Cg*	*Y*—*X*	*X*⋯*Cg*	*Y*⋯*Cg*	*Y*—*X*⋯*Cg*
C1—I1⋯*Cg*3^i^	2.099 (4)	3.975 (2)	6.026 (5)	164.67 (13)

Symmetry code: (i) 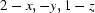.

**Fig. 1.**
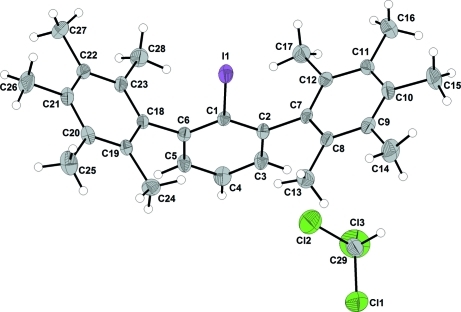
Crystal structure of the title compound with labelling and displacement ellipsoids of C, Cl and I atoms drawn at 25% probability level.

**Fig. 2.**
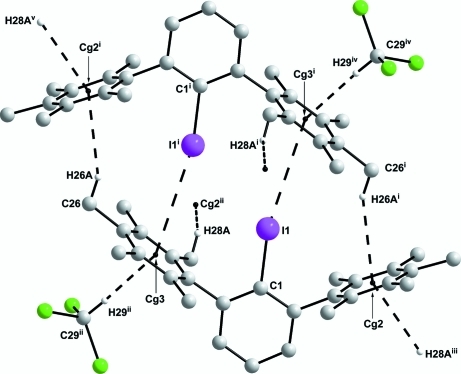
Intermolecular C—H···π and C—I···π interactions in the structure of the title compound. Symmetry codes: (i) 2 - x,-y,1 - z; (ii) 1/2 + x,1/2 - y,1/2 + z; (iii) -1/2 + x,1/2 - y,-1/2 + z; (iv) 3/2 - x, -1/2 + y, 1/2 - z; (v) 5/2 - x, -1/2 + y, 3/2 - z. Cg2 and Cg3 are the centroids of the benzene rings C7–C12 and C18–C23, respectively.

## supplementary crystallographic information

### Comment

*m*-Terphenyl substituents are sterrically crowded ligands used in stabilizing labile bonds and unusual geometries (Power, 2004).

We report herein the crystal structure of the title compound. The synthesis and the spectroscopic characterization of 2'-iodo-2,2'',3,3'',4,4'',5,5'',6,6''-decamethyl-1,1':3',1''-terphenyl were previously reported (Hino *et al.*, 2005; Duttwyler *et al.*, 2008).

The crystal structure (Fig. 1) of the title compound is similar to the structures of other 2,6-diarylphenyliodides. The C—I bond length [2.099 (4) Å] is slightly smaller than those found in 2,6-(2,4,6 - *i*Pr_3_C_6_H_2_)_2_C_6_H_3_I [2.102 (6) Å] (Twamley *et al.*, 2000), 2,6-Ph_2_C_6_H_3_I [2.122 (4) Å] (Niemeyer, 1998), and 2,6-Mes_2_C_6_H_3_I [2.102 (5) Å] (Zakharov *et al.*, 2003).

Similar to the other 2,6-diarylphenyliodides, the dihedral angles between the flanking groups and the central benzene ring are close to 90° (Niemeyer, 1998; Twamley *et al.*, 2000; Zakharov *et al.*, 2003). This arrangement permits the presence of an intramolecular I···*Cg* contact [3.955 (1) Å]. The I···*Cg* interaction is reflected in the difference [Δ = 2.9°] at the C1 bonding angles.

In the crystal structure symmetry related molecules are linked into dimers through C—I···π interactions (Table 1 and Fig. 2). In addition there are C—H···π interactions (Table 2 and Fig. 2) that link the dimeric units and the solvent molecules into a three dimensional network.

### Experimental

The synthesis of 2'-iodo-2,2'',3,3'',4,4'',5,5'',6,6''-decamethyl-1,1':3',1''-terphenyl was carried out according to a previously described method (Hino *et al.*, 2005). Crystals of the title compound were obtained by slow evaporation of the solvent from a solution of 2'-iodo-2,2'',3,3'',4,4'',5,5'',6,6''-decamethyl-1,1':3',1''-terphenyl in chloroform.

### Refinement

Hydrogen atoms were placed in calculated positions with isotropic thermal parameters set at 1.2 times the carbon atoms directly attached for aromatic and methine hydrogen atoms and 1.5 for hydrogen atoms of the methyl groups. Methyl hydrogen atoms were allowed to rotate but not to tip.

### Crystal data

**Table:**

C_28_H_33_I·CHCl_3_	*F*(000) = 1248
*M**_r_* = 615.81	*D*_x_ = 1.415 Mg m^−^^3^
Monoclinic, *P*2_1_/*n*	Melting point = 498–497 K
Hall symbol: -P 2yn	Mo *K*α radiation, λ = 0.71073 Å
*a* = 12.0294 (10) Å	Cell parameters from 3032 reflections
*b* = 16.0651 (13) Å	θ = 2.3–19.9°
*c* = 15.3762 (12) Å	µ = 1.40 mm^−^^1^
β = 103.385 (1)°	*T* = 297 K
*V* = 2890.8 (4) Å^3^	Block, colourless
*Z* = 4	0.32 × 0.28 × 0.26 mm

### Data collection

**Table:**

Bruker SMART CCD area-detector diffractometer	5896 independent reflections
Radiation source: fine-focus sealed tube	4698 reflections with *I* > 2σ(*I*)
graphite	*R*_int_ = 0.056
φ and ω scans	θ_max_ = 26.4°, θ_min_ = 1.9°
Absorption correction: multi-scan (*SADABS*; Bruker, 2000)	*h* = −15→15
*T*_min_ = 0.663, *T*_max_ = 0.712	*k* = −20→20
22910 measured reflections	*l* = −19→19

### Refinement

**Table:**

Refinement on *F*^2^	Primary atom site location: structure-invariant direct methods
Least-squares matrix: full	Secondary atom site location: difference Fourier map
*R*[*F*^2^ > 2σ(*F*^2^)] = 0.066	Hydrogen site location: inferred from neighbouring sites
*wR*(*F*^2^) = 0.137	H-atom parameters constrained
*S* = 1.13	*w* = 1/[σ^2^(*F*_o_^2^) + (0.0465*P*)^2^ + 3.6212*P*] where *P* = (*F*_o_^2^ + 2*F*_c_^2^)/3
5896 reflections	(Δ/σ)_max_ = 0.031
308 parameters	Δρ_max_ = 0.83 e Å^−^^3^
0 restraints	Δρ_min_ = −0.68 e Å^−^^3^

### Special details

**Table:**

Geometry. All s.u.'s (except the s.u. in the dihedral angle between two l.s. planes) are estimated using the full covariance matrix. The cell s.u.'s are taken into account individually in the estimation of s.u.'s in distances, angles and torsion angles; correlations between s.u.'s in cell parameters are only used when they are defined by crystal symmetry. An approximate (isotropic) treatment of cell s.u.'s is used for estimating s.u.'s involving l.s. planes.
Refinement. Refinement of *F*^2^ against ALL reflections. The weighted *R*-factor *wR* and goodness of fit *S* are based on *F*^2^, conventional *R*-factors *R* are based on *F*, with *F* set to zero for negative *F*^2^. The threshold expression of *F*^2^ > 2σ(*F*^2^) is used only for calculating *R*-factors(gt) *etc*. and is not relevant to the choice of reflections for refinement. *R*-factors based on *F*^2^ are statistically about twice as large as those based on *F*, and *R*- factors based on ALL data will be even larger.

### Fractional atomic coordinates and isotropic or equivalent isotropic displacement parameters (Å^2^)

**Table:**

	*x*	*y*	*z*	*U*_iso_*/*U*_eq_	
C1	0.7505 (4)	0.1344 (3)	0.4780 (3)	0.0364 (11)	
C2	0.6846 (4)	0.1920 (3)	0.4197 (3)	0.0383 (11)	
C3	0.6011 (5)	0.2346 (3)	0.4498 (3)	0.0499 (13)	
H3	0.5556	0.273	0.4122	0.06*	
C4	0.5835 (5)	0.2216 (4)	0.5341 (4)	0.0570 (15)	
H4	0.5278	0.2518	0.5535	0.068*	
C5	0.6487 (5)	0.1637 (3)	0.5897 (3)	0.0490 (13)	
H5	0.6354	0.1544	0.6461	0.059*	
C6	0.7338 (4)	0.1191 (3)	0.5631 (3)	0.0377 (11)	
C7	0.6986 (4)	0.2074 (3)	0.3265 (3)	0.0377 (11)	
C8	0.6370 (4)	0.1577 (3)	0.2572 (3)	0.0422 (12)	
C9	0.6476 (4)	0.1740 (3)	0.1698 (3)	0.0476 (13)	
C10	0.7155 (5)	0.2388 (3)	0.1525 (3)	0.0493 (13)	
C11	0.7780 (4)	0.2865 (3)	0.2222 (3)	0.0473 (12)	
C12	0.7713 (4)	0.2701 (3)	0.3103 (3)	0.0422 (12)	
C13	0.5589 (6)	0.0903 (4)	0.2764 (4)	0.0680 (17)	
H13A	0.4823	0.1009	0.2431	0.102*	
H13B	0.5607	0.0898	0.3391	0.102*	
H13C	0.5839	0.0374	0.2592	0.102*	
C14	0.5810 (7)	0.1203 (5)	0.0945 (4)	0.084 (2)	
H14A	0.6145	0.1244	0.0438	0.125*	
H14B	0.5031	0.139	0.0782	0.125*	
H14C	0.5831	0.0634	0.1139	0.125*	
C15	0.7196 (6)	0.2594 (5)	0.0568 (4)	0.084 (2)	
H15A	0.6584	0.2314	0.0163	0.127*	
H15B	0.7914	0.2415	0.046	0.127*	
H15C	0.7117	0.3184	0.0476	0.127*	
C16	0.8538 (6)	0.3562 (4)	0.2035 (5)	0.079 (2)	
H16A	0.813	0.3886	0.1538	0.118*	
H16B	0.9211	0.3331	0.1895	0.118*	
H16C	0.8753	0.3911	0.2553	0.118*	
C17	0.8413 (5)	0.3199 (4)	0.3880 (4)	0.0681 (17)	
H17A	0.8268	0.2997	0.443	0.102*	
H17B	0.8204	0.3776	0.3807	0.102*	
H17C	0.9211	0.3139	0.3893	0.102*	
C18	0.8017 (4)	0.0550 (3)	0.6229 (3)	0.0368 (11)	
C19	0.7593 (4)	−0.0266 (3)	0.6220 (3)	0.0396 (11)	
C20	0.8242 (5)	−0.0875 (3)	0.6762 (3)	0.0468 (13)	
C21	0.9318 (5)	−0.0657 (3)	0.7299 (3)	0.0489 (13)	
C22	0.9726 (4)	0.0152 (3)	0.7312 (3)	0.0459 (12)	
C23	0.9068 (4)	0.0763 (3)	0.6789 (3)	0.0447 (12)	
C24	0.6463 (5)	−0.0464 (4)	0.5600 (4)	0.0608 (16)	
H24A	0.6358	−0.1056	0.5565	0.091*	
H24B	0.645	−0.0248	0.5015	0.091*	
H24C	0.5859	−0.0214	0.5822	0.091*	
C25	0.7790 (6)	−0.1748 (3)	0.6782 (4)	0.076 (2)	
H25A	0.6995	−0.176	0.6484	0.114*	
H25B	0.7879	−0.1924	0.7391	0.114*	
H25C	0.8208	−0.2117	0.6483	0.114*	
C26	1.0042 (6)	−0.1318 (4)	0.7865 (4)	0.078 (2)	
H26A	1.0682	−0.1451	0.7616	0.117*	
H26B	0.9591	−0.1809	0.7876	0.117*	
H26C	1.0314	−0.1113	0.8463	0.117*	
C27	1.0888 (5)	0.0364 (5)	0.7901 (4)	0.0730 (18)	
H27A	1.1433	−0.0051	0.7828	0.109*	
H27B	1.0837	0.038	0.8515	0.109*	
H27C	1.1128	0.0899	0.7733	0.109*	
C28	0.9492 (6)	0.1650 (4)	0.6799 (4)	0.0728 (19)	
H28A	1.0236	0.1655	0.6671	0.109*	
H28B	0.9536	0.189	0.7378	0.109*	
H28C	0.8973	0.1969	0.6354	0.109*	
I1	0.87935 (3)	0.06968 (3)	0.43456 (2)	0.05850 (16)	
C29	0.2774 (6)	0.4708 (4)	0.3696 (4)	0.0684 (17)	
H29	0.2996	0.4802	0.313	0.082*	
Cl1	0.14519 (19)	0.41957 (14)	0.34585 (16)	0.1030 (7)	
Cl2	0.3802 (2)	0.40834 (19)	0.43583 (18)	0.1351 (10)	
Cl3	0.2678 (3)	0.56550 (15)	0.4179 (2)	0.1352 (10)	

### Atomic displacement parameters (Å^2^)

**Table:**

	*U*^11^	*U*^22^	*U*^33^	*U*^12^	*U*^13^	*U*^23^
C1	0.037 (3)	0.036 (3)	0.036 (2)	0.008 (2)	0.008 (2)	0.002 (2)
C2	0.046 (3)	0.038 (3)	0.030 (2)	0.002 (2)	0.006 (2)	0.000 (2)
C3	0.057 (3)	0.048 (3)	0.044 (3)	0.021 (3)	0.012 (3)	0.006 (2)
C4	0.065 (4)	0.056 (4)	0.055 (3)	0.024 (3)	0.024 (3)	−0.005 (3)
C5	0.059 (3)	0.056 (3)	0.035 (3)	0.008 (3)	0.016 (2)	0.002 (2)
C6	0.040 (3)	0.036 (3)	0.036 (3)	0.000 (2)	0.006 (2)	0.001 (2)
C7	0.037 (3)	0.041 (3)	0.033 (2)	0.014 (2)	0.005 (2)	0.007 (2)
C8	0.039 (3)	0.047 (3)	0.037 (3)	0.007 (2)	0.002 (2)	0.006 (2)
C9	0.047 (3)	0.053 (3)	0.039 (3)	0.011 (3)	0.004 (2)	0.000 (2)
C10	0.060 (3)	0.054 (3)	0.036 (3)	0.016 (3)	0.016 (3)	0.008 (2)
C11	0.044 (3)	0.049 (3)	0.049 (3)	0.006 (2)	0.012 (2)	0.015 (3)
C12	0.040 (3)	0.041 (3)	0.044 (3)	0.006 (2)	0.007 (2)	0.003 (2)
C13	0.072 (4)	0.064 (4)	0.065 (4)	−0.013 (3)	0.009 (3)	0.006 (3)
C14	0.104 (6)	0.096 (5)	0.044 (3)	−0.010 (4)	0.005 (4)	−0.015 (4)
C15	0.104 (6)	0.109 (6)	0.046 (4)	0.002 (5)	0.028 (4)	0.015 (4)
C16	0.084 (5)	0.080 (5)	0.077 (5)	−0.008 (4)	0.028 (4)	0.025 (4)
C17	0.067 (4)	0.072 (4)	0.061 (4)	−0.011 (3)	0.006 (3)	0.003 (3)
C18	0.039 (3)	0.040 (3)	0.031 (2)	0.004 (2)	0.008 (2)	0.007 (2)
C19	0.047 (3)	0.044 (3)	0.029 (2)	0.000 (2)	0.011 (2)	0.002 (2)
C20	0.067 (4)	0.038 (3)	0.039 (3)	0.003 (2)	0.018 (3)	0.002 (2)
C21	0.057 (3)	0.055 (3)	0.038 (3)	0.020 (3)	0.017 (2)	0.011 (2)
C22	0.042 (3)	0.062 (4)	0.035 (3)	0.004 (2)	0.010 (2)	0.005 (2)
C23	0.049 (3)	0.049 (3)	0.033 (2)	−0.003 (2)	0.004 (2)	0.004 (2)
C24	0.057 (4)	0.060 (4)	0.063 (4)	−0.012 (3)	0.010 (3)	−0.006 (3)
C25	0.119 (6)	0.037 (3)	0.070 (4)	−0.006 (3)	0.019 (4)	0.005 (3)
C26	0.079 (5)	0.078 (5)	0.073 (4)	0.032 (4)	0.008 (4)	0.028 (4)
C27	0.049 (4)	0.103 (5)	0.059 (4)	0.005 (3)	−0.003 (3)	0.011 (4)
C28	0.074 (4)	0.065 (4)	0.070 (4)	−0.023 (3)	−0.002 (3)	0.008 (3)
I1	0.0572 (2)	0.0742 (3)	0.0485 (2)	0.03317 (19)	0.02111 (17)	0.01532 (19)
C29	0.072 (4)	0.068 (4)	0.070 (4)	0.006 (3)	0.027 (3)	0.014 (3)
Cl1	0.0842 (14)	0.1114 (17)	0.1078 (16)	−0.0161 (12)	0.0108 (12)	0.0079 (13)
Cl2	0.0936 (17)	0.165 (3)	0.144 (2)	0.0425 (16)	0.0228 (16)	0.0602 (19)
Cl3	0.160 (3)	0.1012 (18)	0.150 (2)	−0.0052 (16)	0.048 (2)	−0.0496 (16)

### Geometric parameters (Å, °)

**Table:**

C1—C6	1.392 (6)	C16—H16C	0.96
C1—C2	1.400 (6)	C17—H17A	0.96
C1—I1	2.099 (4)	C17—H17B	0.96
C2—C3	1.381 (6)	C17—H17C	0.96
C2—C7	1.501 (6)	C18—C23	1.398 (7)
C3—C4	1.377 (7)	C18—C19	1.406 (7)
C3—H3	0.93	C19—C20	1.400 (7)
C4—C5	1.379 (7)	C19—C24	1.503 (7)
C4—H4	0.93	C20—C21	1.408 (8)
C5—C6	1.386 (7)	C20—C25	1.507 (7)
C5—H5	0.93	C21—C22	1.389 (7)
C6—C18	1.492 (6)	C21—C26	1.514 (7)
C7—C12	1.394 (7)	C22—C23	1.393 (7)
C7—C8	1.398 (7)	C22—C27	1.518 (8)
C8—C9	1.403 (7)	C23—C28	1.513 (7)
C8—C13	1.507 (7)	C24—H24A	0.96
C9—C10	1.386 (7)	C24—H24B	0.96
C9—C14	1.516 (8)	C24—H24C	0.96
C10—C11	1.389 (7)	C25—H25A	0.96
C10—C15	1.521 (7)	C25—H25B	0.96
C11—C12	1.401 (7)	C25—H25C	0.96
C11—C16	1.512 (8)	C26—H26A	0.96
C12—C17	1.520 (7)	C26—H26B	0.96
C13—H13A	0.96	C26—H26C	0.96
C13—H13B	0.96	C27—H27A	0.96
C13—H13C	0.96	C27—H27B	0.96
C14—H14A	0.96	C27—H27C	0.96
C14—H14B	0.96	C28—H28A	0.96
C14—H14C	0.96	C28—H28B	0.96
C15—H15A	0.96	C28—H28C	0.96
C15—H15B	0.96	C29—Cl3	1.708 (7)
C15—H15C	0.96	C29—Cl2	1.729 (7)
C16—H16A	0.96	C29—Cl1	1.753 (7)
C16—H16B	0.96	C29—H29	0.98
			
C6—C1—C2	122.3 (4)	C12—C17—H17A	109.5
C6—C1—I1	119.4 (3)	C12—C17—H17B	109.5
C2—C1—I1	118.3 (3)	H17A—C17—H17B	109.5
C3—C2—C1	117.5 (4)	C12—C17—H17C	109.5
C3—C2—C7	119.3 (4)	H17A—C17—H17C	109.5
C1—C2—C7	123.2 (4)	H17B—C17—H17C	109.5
C4—C3—C2	121.5 (5)	C23—C18—C19	120.6 (4)
C4—C3—H3	119.3	C23—C18—C6	119.9 (4)
C2—C3—H3	119.3	C19—C18—C6	119.5 (4)
C3—C4—C5	119.8 (5)	C20—C19—C18	119.7 (5)
C3—C4—H4	120.1	C20—C19—C24	121.9 (5)
C5—C4—H4	120.1	C18—C19—C24	118.5 (4)
C4—C5—C6	121.2 (5)	C19—C20—C21	119.0 (5)
C4—C5—H5	119.4	C19—C20—C25	120.5 (5)
C6—C5—H5	119.4	C21—C20—C25	120.4 (5)
C5—C6—C1	117.7 (4)	C22—C21—C20	121.0 (5)
C5—C6—C18	120.8 (4)	C22—C21—C26	119.6 (5)
C1—C6—C18	121.5 (4)	C20—C21—C26	119.4 (5)
C12—C7—C8	121.3 (4)	C21—C22—C23	120.0 (5)
C12—C7—C2	120.2 (4)	C21—C22—C27	119.5 (5)
C8—C7—C2	118.5 (4)	C23—C22—C27	120.5 (5)
C7—C8—C9	118.3 (5)	C22—C23—C18	119.7 (5)
C7—C8—C13	120.3 (5)	C22—C23—C28	120.9 (5)
C9—C8—C13	121.3 (5)	C18—C23—C28	119.5 (5)
C10—C9—C8	120.8 (5)	C19—C24—H24A	109.5
C10—C9—C14	120.8 (5)	C19—C24—H24B	109.5
C8—C9—C14	118.4 (5)	H24A—C24—H24B	109.5
C9—C10—C11	120.3 (5)	C19—C24—H24C	109.5
C9—C10—C15	120.2 (5)	H24A—C24—H24C	109.5
C11—C10—C15	119.6 (5)	H24B—C24—H24C	109.5
C10—C11—C12	120.0 (5)	C20—C25—H25A	109.5
C10—C11—C16	120.3 (5)	C20—C25—H25B	109.5
C12—C11—C16	119.7 (5)	H25A—C25—H25B	109.5
C7—C12—C11	119.2 (5)	C20—C25—H25C	109.5
C7—C12—C17	119.8 (5)	H25A—C25—H25C	109.5
C11—C12—C17	121.0 (5)	H25B—C25—H25C	109.5
C8—C13—H13A	109.5	C21—C26—H26A	109.5
C8—C13—H13B	109.5	C21—C26—H26B	109.5
H13A—C13—H13B	109.5	H26A—C26—H26B	109.5
C8—C13—H13C	109.5	C21—C26—H26C	109.5
H13A—C13—H13C	109.5	H26A—C26—H26C	109.5
H13B—C13—H13C	109.5	H26B—C26—H26C	109.5
C9—C14—H14A	109.5	C22—C27—H27A	109.5
C9—C14—H14B	109.5	C22—C27—H27B	109.5
H14A—C14—H14B	109.5	H27A—C27—H27B	109.5
C9—C14—H14C	109.5	C22—C27—H27C	109.5
H14A—C14—H14C	109.5	H27A—C27—H27C	109.5
H14B—C14—H14C	109.5	H27B—C27—H27C	109.5
C10—C15—H15A	109.5	C23—C28—H28A	109.5
C10—C15—H15B	109.5	C23—C28—H28B	109.5
H15A—C15—H15B	109.5	H28A—C28—H28B	109.5
C10—C15—H15C	109.5	C23—C28—H28C	109.5
H15A—C15—H15C	109.5	H28A—C28—H28C	109.5
H15B—C15—H15C	109.5	H28B—C28—H28C	109.5
C11—C16—H16A	109.5	Cl3—C29—Cl2	111.9 (4)
C11—C16—H16B	109.5	Cl3—C29—Cl1	111.1 (4)
H16A—C16—H16B	109.5	Cl2—C29—Cl1	110.0 (4)
C11—C16—H16C	109.5	Cl3—C29—H29	107.9
H16A—C16—H16C	109.5	Cl2—C29—H29	107.9
H16B—C16—H16C	109.5	Cl1—C29—H29	107.9
			
C6—C1—C2—C3	0.3 (7)	C2—C7—C12—C11	−176.4 (4)
I1—C1—C2—C3	−179.4 (4)	C8—C7—C12—C17	−176.9 (5)
C6—C1—C2—C7	−177.9 (4)	C2—C7—C12—C17	3.3 (7)
I1—C1—C2—C7	2.5 (6)	C10—C11—C12—C7	−2.0 (7)
C1—C2—C3—C4	0.5 (8)	C16—C11—C12—C7	178.1 (5)
C7—C2—C3—C4	178.8 (5)	C10—C11—C12—C17	178.3 (5)
C2—C3—C4—C5	−1.3 (9)	C16—C11—C12—C17	−1.5 (8)
C3—C4—C5—C6	1.3 (9)	C5—C6—C18—C23	−94.7 (6)
C4—C5—C6—C1	−0.5 (8)	C1—C6—C18—C23	87.5 (6)
C4—C5—C6—C18	−178.3 (5)	C5—C6—C18—C19	85.6 (6)
C2—C1—C6—C5	−0.3 (7)	C1—C6—C18—C19	−92.2 (6)
I1—C1—C6—C5	179.3 (4)	C23—C18—C19—C20	−1.4 (7)
C2—C1—C6—C18	177.5 (4)	C6—C18—C19—C20	178.3 (4)
I1—C1—C6—C18	−2.8 (6)	C23—C18—C19—C24	−179.5 (5)
C3—C2—C7—C12	89.3 (6)	C6—C18—C19—C24	0.2 (7)
C1—C2—C7—C12	−92.5 (6)	C18—C19—C20—C21	−0.8 (7)
C3—C2—C7—C8	−90.5 (6)	C24—C19—C20—C21	177.2 (5)
C1—C2—C7—C8	87.7 (6)	C18—C19—C20—C25	178.3 (5)
C12—C7—C8—C9	−1.7 (7)	C24—C19—C20—C25	−3.7 (8)
C2—C7—C8—C9	178.1 (4)	C19—C20—C21—C22	1.5 (7)
C12—C7—C8—C13	−179.9 (5)	C25—C20—C21—C22	−177.6 (5)
C2—C7—C8—C13	−0.2 (7)	C19—C20—C21—C26	−178.1 (5)
C7—C8—C9—C10	−1.4 (7)	C25—C20—C21—C26	2.8 (8)
C13—C8—C9—C10	176.8 (5)	C20—C21—C22—C23	0.1 (7)
C7—C8—C9—C14	−180.0 (5)	C26—C21—C22—C23	179.7 (5)
C13—C8—C9—C14	−1.8 (8)	C20—C21—C22—C27	179.8 (5)
C8—C9—C10—C11	2.7 (8)	C26—C21—C22—C27	−0.6 (8)
C14—C9—C10—C11	−178.7 (5)	C21—C22—C23—C18	−2.3 (7)
C8—C9—C10—C15	−175.7 (5)	C27—C22—C23—C18	178.0 (5)
C14—C9—C10—C15	2.8 (8)	C21—C22—C23—C28	179.5 (5)
C9—C10—C11—C12	−1.0 (8)	C27—C22—C23—C28	−0.2 (8)
C15—C10—C11—C12	177.5 (5)	C19—C18—C23—C22	3.0 (7)
C9—C10—C11—C16	178.8 (5)	C6—C18—C23—C22	−176.7 (4)
C15—C10—C11—C16	−2.7 (8)	C19—C18—C23—C28	−178.7 (5)
C8—C7—C12—C11	3.4 (7)	C6—C18—C23—C28	1.5 (7)

### Hydrogen-bond geometry (Å, °)

**Table:**

Cg2 and C3 are the centroids of the C7–C12 and C18–C23 benzene rings, respectively.

**Table:**

*D*—H···*A*	*D*—H	H···*A*	*D*···*A*	*D*—H···*A*
C26—H26A···Cg2^i^	0.96	3.86 (1)	2.97	155
C28—H28A···Cg2^ii^	0.96	3.53 (1)	2.87	127
C29—H29···Cg3^iii^	0.98	3.42 (1)	2.44	177

Symmetry codes: (i) −*x*+2, −*y*, −*z*+1; (ii) *x*+1/2, −*y*+1/2, *z*+1/2; (iii) *x*+1/2, −*y*+1/2, *z*−1/2.

### Table 2 C—I···π interactions (Å, °)

Cg3 is the centroid of the  C18–C23 benzene ring.

**Table:**

*Y*—*X*···Cg	*Y*—*X*	*X*···Cg	*Y*···Cg	*Y*—*X*···Cg
C1—I1···Cg3^i^	2.099 (4)	3.975 (2)	6.026 (5)	164.67 (13)

Symmetry code: (i) 2-x, -y, 1-z.
